# Umbilical cord blood banking and its interruptions: notes from Chennai, India

**DOI:** 10.1080/03085147.2013.772759

**Published:** 2013-09-09

**Authors:** Sarah Hodges

**Keywords:** umbilical cord blood banking, stem cell, Chennai, India

## Abstract

What is the relationship between globalization and the body? In both scholarly discourse and everyday practice, the relationship between globalization and the body has been understood through the idea of accumulation – the intensified and interlinked pursuit of health and wealth. Through an examination of private umbilical cord blood banking in south India, this paper investigates the interplay of accumulation *and* its interruption in Indians' uptake of this practice. Although Chennai-based bankers, doctors and parents explain the recent surge in popularity for cord blood banking in the city in terms of interlinked financial and familial accumulation, investment and security, Indians' cultural and religious conceptions of the latent power of the placenta and cord often work *against* cord blood banking marketers' neat account of twinned economic and familial accumulation. Analytic attention to interruption highlights the tensions within and precariousness of the accumulation at the heart of the relationship between globalization and the body.

What is the relationship between globalization and the body? In the late 1990s, David Harvey remarked that, despite that decade's efflorescence of scholarship on ‘the body’ and a simultaneous scholarly preoccupation with globalization, this had not led to systematic attempts to link discourses on the body with the economic and cultural practices of globalization (Harvey, 1998). Fifteen years on, the emergence of global markets for medical goods and services – such as assisted reproductive technologies, organ transplantation and regenerative medicine – has created large-scale convergences of bodily practices among the rich the world over.

As if in response to Harvey's concerns, there is now much work that traces the making of a globalized, medicalized body (for example, Bharadwaj & Glasner, 2009; Cohen, 2011; Inhorn, 2003; Roberts, 2007; Waldby & Cooper, 2010). One cluster of work uses ‘globalization’ to refer to forms of intensified trade across larger distances and has investigated the growth in global networks of commerce for biomedical research and biomedical therapeutics (Bharadwaj & Glasner, 2009; Cohen, 2005; Dickenson, 2008; Parry, 2004; Scheper-Hughes, 2000; Thacker, 2005). This work has illuminated how the new ease of travel for larger numbers of moneyed patients, in conjunction with the emergence of new technologies (such as immunosuppressant transplant drugs), has allowed ever greater numbers of practitioners and patients to exploit the significant national differences in the relative cost of medical care and medical ‘supplies’ (such as ova, surrogate/gestational mothers or kidneys).

Another cluster of work uses ‘globalization’ to refer to neoliberalism (economic policies of market liberalization alongside states' and corporations' efforts to lower ‘barriers’ to trade in pursuit of economic growth). Giving particular attention to medical biotechnologies, this work points out the interlinked pursuit of ‘growth’ and ‘life’ that can be observed over the past few decades across (1) claims made regarding the potential benefits of neoliberal policies and (2) claims made regarding the regenerative potential of stem cell therapeutics (Cooper, 2008; Franklin, 2005; Sunder Rajan, 2006; Waldby & Cooper, 2010; Waldby & Mitchell, 2006). These analyses have gone far to outline the interlinked financial, research, marketing and practical therapeutic strategies of new medical bio-technologies the world over as they emerged alongside and in dialogue with new economic thought and practice.

Taken together, this work points to how, in both scholarly discourse and everyday practice, ‘accumulation’ is central to the relationship between globalization and the body. In other words, the body has become enmeshed in multiple, and often novel, practices for the extraction and accumulation of wealth. In particular, these practices of accumulation are manifest within an intensified and interlinked pursuit of health and wealth. The rise of globalization has occasioned the seeking out and creation of new revenue streams – for and of the body – that were earlier either technically impossible and/or morally/culturally forbidden. Yet, particularly in attempts to understand new medical biotechnologies, I worry that, in attending to this bigger story of accumulation and its corollary ‘flows’, we have neglected the many interruptions that pepper these practices. If ‘accumulation’ lies at the heart of the many claims regarding the relationship between globalization and the body, then its ‘interruption’ poses awkward, and ultimately productive, epistemic and methodological questions.

By way of addressing these issues, this paper examines the interplay of accumulation *and* interruption in Indians' uptake of the relatively new practice of private umbilical cord blood banking – in which the stem cells found in umbilical cord blood are stored by a baby's family as a form of biological ‘insurance’. It is based on conversations I had over the course of several years (2007–10) with members of Chennai's cord blood communities – in the homes, offices and alleyways of private and public cord blood bankers, marketers, obstetricians, hematologists, technicians, clients, donors and other parents. To anticipate the argument somewhat, it is certainly the case that Chennai-based bankers, doctors and parents explain the recent surge in popularity for cord blood banking in the city in terms of interlinked financial and familial accumulation, investment and security. Additionally, those in the city's cord blood communities stress not only the potential therapeutic value of banked cord blood, but also its linked moral and economic value accruing from the reclamation of ‘waste’. Despite this, time and again in my conversations, additional registers of value figured prominently in Chennai residents' accounting for the power and potential of cord blood. Indeed, Indians' cultural and religious conceptions of the latent power of the placenta and cord often worked against and interrupted cord blood banking marketers' neat narratives of security, waste redemption and twinned economic and familial accumulation. Attention to these interruptions – occasions where extraction and accumulation do not happen or are thrown into question – matters because it highlights the precariousness of accumulation as discourse and practice under globalization.

## Why is cord blood special?

The stem cells found in umbilical cord blood are the object of scientific and commercial interest because of their *regenerative* properties. For my purposes, the banking of these cells in Chennai matters because it highlights key elements of accumulation and interruption at the heart of the relationship between globalization and the body. Cord blood banking provides one example of the complex set of practices that enmesh the body and use it and its capacities as an economic resource. In particular, cord blood banking brings together (1) new technologies, (2) new horizons of medical treatment and (3) new revenue streams. Although stem cells have been an object of scientific research since the late nineteenth century, therapeutic applications of stem cell science, particularly in regenerative medicine, emerged only during the final decades of the twentieth century (Maehle, 2011). In the 1990s, despite research that suggested substantial potential for stem cell treatments to regenerate and thereby replace a patient's damaged cells, much of this work used embryonic cells. Embryonic stem cell research and treatments encountered substantial opposition from Christian faith communities, particularly within the US, on the grounds that they both destroyed and commodified life (Cooper, 2008, pp. 152–75).

In contrast, stem cells harvested from post-partum umbilical cord blood offered research material and commercial and therapeutic possibilities largely unburdened by the popular controversies that dogged embryonic stem cells.^1^ Further, the cells found in umbilical cord blood presented new commercial possibilities *latent in every birth* for harvesting and private banking. Since the 1980s, stem cells from umbilical cord blood have been used to treat blood disorders (e.g. anaemia, leukaemia and thalassemias).^2^ By the mid-1990s, companies across the globe started to market services for the private banking of stem cells taken from cord and cord blood, in which only the family who banked the cells had access to them (Dickenson, 2008; Technopolis, 2009; Waldby & Mitchell, 2006, pp. 110–30).^3^ Around this time, ‘public’ cord blood banks for donated samples also began to appear (Busby & Martin, 2006).

Prospective private clients are marketed banking services on the grounds that, even though banked cells might not be needed immediately, the child whose cells are banked privately may be able to use them later in life, when, marketers suggest, even more stem cell treatments may have been discovered. ^4^ This is, however, a somewhat problematic claim even if further treatments should materialize in future. In most conditions currently treatable with cord blood cells, this child's *own* cells are of no use (because the banked cells would have the same problem). Further, the amount of stem cells generally available from an umbilical cord blood unit (*c*. 60 ml) is adequate only for treating a child up to the age of about 8 or 9. After that, the treatments that are currently available require additional cells to be sourced. This causes patients to incur further costs and runs the risk that a match may not be found. Banks also promote the idea that banked cord blood could be used to treat a member of the extended family. This claim is also problematic because, although there is a 25 per cent chance that a sibling could provide a workable match, it remains unlikely that other relatives might.^5^ Nevertheless, bankers' claims for the extended family currency of cord blood are bolstered when considered alongside current speculative claims for future stem cell treatments. These treatments include those for diabetes and heart disease – conditions that may cloud the horizon of older generations in a family. In short, although private banking services are marketed to families in terms of ‘securing your baby's health’ with the corollary suggestion that it may secure a baby's grandparents' health as well, it is not clear whether the security gained in private cord blood banking is on a collective familial or an individual level.

## Why Chennai, India?

A coastal city in south-east India, Chennai (formerly Madras) is the state capital of Tamil Nadu and is the fourth largest city in India. A city famed throughout the twentieth century for its high standard of health care (and, more recently, for its high-tech, high-priced ‘corporate’ private hospitals), cord blood bankers in Chennai are marketing the city as ‘the next global hub’ for stem cell research and treatment (*ExpressPharma*, 2008; Knowledge@Wharton, 2010). Indeed, since banking began there in 2004, Chennai has emerged as one of India's foremost centres for applied cord blood therapeutics and cord blood storage.^6^ All of India's major cord blood banking firms compete in Chennai for the city's growing clientele. In conversation, Chennai's prominent obstetricians estimated that between one-quarter and one-third of their clients bank their cord blood and that this proportion continues to increase at a brisk pace.

Cord blood banking became available in India during a recent – and celebrated – chapter of the country's history which has seen significant economic growth alongside new policies of market liberalization (Corbridge & Harriss, 2000; Gupta & Sivaramakrishnan, 2011; Harriss-White, 2004; Ruparelia *et al*., 2011). These market reforms have largely been credited with laying the groundwork for India's recent, if uneven, economic successes. They have also been credited with creating new areas of economic growth for India – including the corporate health care and biotechnology sectors (Baru, 1998; Jeffery & Jeffery 2008; Nandraj *et al*., 2001; Rao, 1999, 2009). Chennai's cord blood banking scene bears the hallmarks of post-liberalization India in a number of ways.

One way that cord blood banking articulates post-liberalization India's character is through cord blood banking's corporate health-care model. Despite becoming available more than a decade after banking was first available in the US or UK, cord blood banking has expanded rapidly in India using the model of offering a global standard in high-priced specialist services for those who can afford to pay. In light of this, as one scholar has noted, services like cord blood banking in post-liberalization India point to how the ‘most profound effect [of liberalization] could be on India's sense of itself and its place in the world’ (Mehta, 2007, pp. 186–7). This newfound sense of entitlement informs affluent Indians' attitudes regarding consumer choice. In cord blood banking, this translates to articulations of national pride and ‘arrival’ embedded within marketing strategies for high-quality, high-tech and high-priced medical services. As one founder of India's corporate health care industry explained to me: ‘No one should have to travel abroad for high quality treatment. India can now provide for her own’.

Another manifestation of post-liberalization India in cord blood banking is to be seen in banks' role within India's capital budgeting strategies for health-care services and medical research. In the opening decades of the twentieth century, health care – and science in general – were seen to hold the key to solving India's poverty. A century later, health care in India is seen as a driver of economic growth (Hodges & Rao, forthcoming). The idea of high-tech medical care and services as a revenue generator rather than a public service informs much strategic thinking in cord blood banking. India is unlike many countries in that it has one of the world's biggest privately banked collections (second only to the US),^7^ yet offers almost no public banking (Technopolis, 2009).^8^ Cord blood banks – whether public or private – are capital intensive and costly to get up and running. Nevertheless, large private cord blood banks in India, such as ReliCord or LifeCell, have not sought a public listing. These banks tend to be branches of highly capitalized Indian parent companies. For example, ReliCord is a subsidiary of Reliance Industries (India's largest conglomerate) and LifeCell is part of the Shasun group (Shasun is one of the world's largest manufacturers of ibuprofen). The head of research and development at one of Chennai's (and India's) largest private banks explained that the revenue from private banking is intended to underwrite the on-going research into and development of new commercial therapeutic applications for blood stem cells.^9^

Finally, economic growth and market liberalization in India have coincided with a spirit of ‘deregulation’ after what some claimed was an economically stultifying policy of ‘license Raj’ in which government permission had to be obtained for a (comparatively speaking) vast set of business activities (Chatterjee, 2011, pp. 23–4; Sinha, 2011). Indeed, India's liberalization has been singled out as distinctive by virtue of its ‘state-led’ character (Kaviraj, 2011, pp. 43–6). Today, cord blood retail banking and commercial research are far less regulated in India than in other countries (Bharadwaj & Glasner, 2009, pp. 98–106; Salter, 2008, pp. 152–3). The same research and development director referred to above told me how shocked he is when he returns to the US (where he had worked for almost two decades) and sees the comparatively slow pace of work there:

R&D: They are still working with the mouse! They have the best facilities in the world and they are working with *the mouse*! All they can say is: ‘Well, it works in the mouse, it *just might* work in humans’.^10^ Here [in Chennai], we are working with questions of direct applicability. There are such tight regulations there [in the US] regarding what you can and can't store.

SH: So … there is more freedom in Chennai?

R&D: Well, maybe more freedom … or maybe just no rules.

He went on to explain that the research and treatment guidelines and regulations that did exist were generally written by people in industry rather than government, and that government agencies were either uninterested in or unable to keep pace with the changes in the science, observations echoed in existing research (Bharadwaj & Glasner 2009, pp. 104–6; Salter 2008, pp. 150–3).

In a subsequent conversation, I asked this head of research and development if he had come across *The immortal life of Henrietta Lacks*, the new book on HeLa cells (Skloot, 2010).^11^ He replied: ‘Yes. I mean no, I haven't read the book but I know the story – See? All that happened at a time in America when there was almost no regulation! That's what we have now here [in India], too!’

Despite the relatively unregulated status of cord blood banking, research and therapies, the marketing of cord blood banking is highly structured in ways that highlight its utility within broader strategies of biological and financial accumulation – both individual and familial.

## The business of cord blood banking in Chennai: health is wealth and family is for ever

Indian cord blood stem cell bankers offer a variation on the marketing themes that have now become commonplace in cord blood banking the world over – that banking cord blood stem cells is the closest thing to a ‘security blanket’ for expectant families to gift their unborn child. Similar tag-lines can be found in most cord blood banking websites, regardless of national location, such as: ‘Take care! … of the miracle within’ (US); ‘With you for life’ (Canada); ‘The best start in life’ (Australia). However, cord blood banking services in Chennai also seek to tap into what they regard as families' pre-existing proclivities to maximize their interlinked reproductive and economic success. By banking cord blood stem cells, bankers explain, families can mobilize and maximize their collective financial and biological capital. Cord blood stem cell banking marketers' promotional materials and ‘pitch’ speak to a particularly Indian bio-politics that sits comfortably alongside biomedical registers of stem cells' value.

Marketing materials for India's banks and for banks across the globe announce that, while the current value of cord blood stem cells lies in treating childhood blood disorders, additional value may be realized in the future. These materials explain that the value of cord blood stem cell banking lies in harnessing the *potential* value held in cord blood stem cells. Through storing these cells, banks claim, expectant families can protect their unborn child against the perils of the future and its unknown. The main message is that the potential value of banking cord blood stem cells is health itself. But the corollary message is that the full potential of each unborn child's health can be accessed only through applying the techniques and knowledge of modern biomedical science.

Indian cord blood stem cell banks' marketing frames this message of health protection through techno-science alongside its other message: that cord blood stem cell banking is an investment in the financial and biological unit of the traditional Indian joint family. Marketing materials regularly feature portraits of several generations of a family gathered together, framed by joint family-inflected slogans: ‘Safeguard your baby … and generations to come’. These materials point to how cord blood stem cell banking facilitates the continuation of the family line. One cord blood stem cell banker who had previously worked in the US summed up his view of the key difference between banking in the US and banking in India: ‘In the US, they market to the expectant mother. In India, we market to her in-laws’.

This financial and biological investment in the future is authorized by an ideology of protecting the *paramparam* (pedigree). Such an investment sits well within a corollary understanding of the dual function and nature of the so-called traditional joint family in India: that the family perpetuates itself both biologically and financially. Feminist scholars have long pointed out that, across the globe, property is chief among the many things that marriage and the family are best designed to perpetuate (Nair, 1996, pp. 145–213). Given that the bulk of marriages in India are arranged, and brides bring substantial dowries to them, families are well primed to fit cord blood marketers' message within long-standing ideas about how a good family manages its collective biological and financial assets with prudence and efficiency.

Corporate cultures have also begun to extend their entrepreneurial attempts at recruiting and retaining staff by tapping into India's *domestic* corporate approach to managing family assets. This is particularly the case in Chennai's emergent corporate cultures within the rapidly expanding information technology sector. These companies seek to capitalize on their employees' demographics and explain that the ‘average age of employees is about 29 years, when people plan families' (Datta, 2006). One of India's largest cord blood banks has been quick to spot this opportunity. In addition to marketing their services through health-care professionals, this bank has also made arrangements with a number of IT firms. In these arrangements, firms offer cord blood banking as a ‘perk’ within an overall recruitment package designed by companies to impress potential employees in what everyone recognizes as jobs that are highly paid and highly selective, but also characterized by high turnover (Fuller & Narasimhan, 2007). Corporate managers' and cord blood bankers' views coalesce with and build on the popular wisdom: jobs may come and go, but family is for ever and health is wealth.

In cord blood banks' marketing materials and activities, the potential value of the stem cells found in cord blood is regularly contrasted with the waste incurred when cord blood goes unbanked. One marketer recited for me the first sentences of his regular ‘pitch’ for Tamil-medium prospective clients in Chennai: ‘*Thoppukkodi ratham enna enru, teriyuma? Koluntai piranta pirahu ithu perumbalam ‘waste’-a pora vishayam. Athavathu, dust bin-le tookippora vishayam. Ithu oru uyiru-kappathu vishayam* [Do you know what umbilical cord is? After a baby is born, this usually is regarded as waste. That is, this is something that is thrown into the dust bin. This is a life-saving thing]’. Similarly, another cord blood bank's promotional materials begin with the explanation:

The umbilical cord connecting the baby with the mother supports and nourishes the baby for 9 months in the womb. This cord is cut at the time of delivery and discarded as waste after the baby is born. Breakthrough medical research has now shown that the umbilical cord blood and cord tissue are one of the richest sources of stem cells, which have a high potential to treat over 75 serious ailments and many more critical ailments in the future through stem cell therapy.

The message is three-fold: that placenta and cord are ‘waste’ is self-evident, the reclamation of waste into a potentially life-saving substance through cord blood banking is unfettered by any ethical problems, and, by banking, any family can join this moment and movement of scientific progress.

As part of the ‘anyone can do it’ message, cord blood marketing also mobilizes a language of ‘affordability’. When cord blood banking first launched in the city, the service cost approximately Rs 80,000 (between two and three times the cost of the delivery itself at the most prestigious and expensive hospitals or maternity homes).^12^ By 2009, the cost had dropped to Rs 60,000 (now about twice the cost of the most expensive deliveries).^13^ Today, firms marketing banking offer monthly payment plans in addition to their one-off payment option. One marketer spoke of monthly payment plans as central to expanding the client base:

It's not only IT guys [largely regarded as the *nouveau riche* of the city], you have business people, I mean, we cater to the entire middle class. … The affordability also plays a very important role. We are the most affordable bank, compared to the other banks, [we charge] Rs 59,900 for 21 years' [storage], but we customize it. And we have it in equal monthly instalments when you can pay Rs 2,600 for 24 months. So even that Rs 2,600, everyone can manage.

In short, the marketing message of cord blood banking in Chennai makes sense only if one buys into its narratives of linked familial biological and financial investment and accumulation.

## Marketing tactics

In Chennai, private banks put significant resources towards marketing their core message of the potential health value of banking cord blood stem cells. One large bank operates a 24-hour toll-free call centre out of Chennai, where the dozen or so operators on duty in any given shift speak multiple Indian languages. Banks also maintain close links with obstetricians and upscale ‘maternity studios’ in the city. Cord blood banking marketers make informational presentations during the antenatal information sessions and courses that these groups provide.

Cord blood banks' glossy brochures and slick websites feature cinema stars' endorsements and well-produced video testimonials by clients as well as video presentations by experts intended to answer all possible questions. Existing clients are incentivized to recruit new clients with offers of vouchers for beauty treatments at high-profile salons and brand-name children's toys. Private banks also have several chrome and glass-fronted marketing offices around the city's prominent retail areas. Additionally, cord blood banks maintain teams of dedicated marketing staff. Of these cord blood stem cell banking marketers, some visit motherhood exhibitions and spend time in Mothercare and other large maternity chain stores around the city where they greet shoppers and distribute marketing materials.

Nevertheless, the bulk of cord banking recruitment usually begins with frequent, brief interactions with potential clients in clinical settings. One of Chennai's largest private banks employs a team of almost a dozen direct marketers. Half of these employees – called ‘counsellors’ – spend their days in hospitals and maternity-home waiting rooms. They greet pregnant women and distribute marketing materials to them while they are waiting for their check-ups.

In all these contexts, the marketer's brief is to create interest and gather potential contact details to pass onto their colleagues to pursue. One employee explained: ‘We are the brand ambassador of the product. Every day we have to go [and interact with patients waiting to see their obstetrician]. We have to go; we have to generate [interest]. Because I keep on seeing people; only that way we can make the *lead* into a *prospect*’. The marketer's boss jumped in, using me as an example for how to ‘make the lead into a prospect’: ‘So you build a relationship. … Even if [gesturing to me] Sarah is not quite interested to enrol in the fifth month, definitely she will enrol by the eighth [by which time she will have seen our representatives so many times]’. The marketing manager went on to explain that all counsellors are assigned specific hospitals and nursing homes to target and that ‘counsellors are the portfolio managers for their hospitals’. Counsellors also rely on current clients to persuade other patients of the same obstetrician. The marketing manager continued: ‘And if you have registered, and if you are sitting on that side, you may tell that lady [after the counsellor has spoken to her]: “I have registered with this firm … do it: it's *good*.” So that's how it works’.

Cord blood stem cell bankers agreed that their most important ‘concept promotion’ activity was a visit to prospective clients' homes. One marketer explained: ‘We'll meet the patients in hospital and give the pamphlets to the girls [expectant mothers]. We explain a very brief concept and we fix an appointment [for a home visit]. Then we give the appointment to the relationship executive [who will visit them at home]’. Time and again cord blood marketers explained to me how, while they use doctors' practices to ‘generate leads’, the real convincing happens in prospective clients' families' homes. Crucially, these home visits are pitched not only to the expectant parents, but also to the expectant paternal grandparents, who often foot the bill. One head of marketing at one of Chennai's cord blood stem cell banks explained: ‘We have a policy that we will only make a presentation if the *whole* family is there’, by which he meant both the unborn child's parents and its paternal grandparents. In marketers' home visits, contracts are signed and money changes hands. The importance of the home visit within client recruitment is a direct outcome of the importance marketers give to the joint family as the key decision-making unit.

Another cord blood banker who liaises with families emphasized the often intense nature of these visits to families' homes:

[I]f it is going to be a first delivery in the family, *everyone* will be seated. I'll be in the centre to explain because they will be shooting off the questions: whether it's going to affect the baby, us collecting the sample; whether we are taking away the important blood from the baby. I have to explain the things – how it's going to be. I'll be explaining the delivery procedure; what all the collection technician does. This is going to be – actually the placental blood is going to be taken after the clamping of baby and once the baby is removed. So I have to explain the things. So – this is going to be a very crucial situation when I am handling the old age people like father-in-law and mother-in-law.

Conversations with marketers about home visits revealed that they were regularly asked by families to explain both the financial and biomedical prospects of return on the proposed family investment. One marketer explained: ‘Hopefully the joint family [members] will all be there. They will be [asking questions like] how many years the cells can be preserved … what are all the [reasons] for preserving your cells, then what about the company's insurance or financial stability’. In these accounts, cord blood banking marketers tie up their work within the larger and enduring interlinked categories of family, health and wealth. Nevertheless, potential clients' beliefs about the interlinked nature of health and wealth sometimes raised questions about the advisability of banking cord blood.

## The multiple values of umbilical cord

Those who promote cord blood banking are happy to point out how stem cells' medical and financial value coalesces with families' broader strategies of financial and biological accumulation. However, this landscape of value is populated by yet another, extra-scientific quality: that of the supernatural. Within these extra-scientific narratives, cord performs overlapping functions: it remains ‘regenerative’, but is used as a cure for infertility. Similarly, as a different form of ‘insurance’, it is worn by the child in an amulet.^14^ Whether talking with obstetricians, haematologists, cord blood collectors, cord banking marketers, cord blood call centre workers, cord blood banking clients or simply ordinary mothers – many women rehearsed, and at times collapsed, these multiple uses for cord and multiple registers in which cord and placentas are regarded as powerful (Mahalakshmy *et al*., 2008; Santoro, 2011).

Explaining the uses of the umbilical cord in treating infertility, one woman explained:

I have heard that if there was a woman who could not conceive, she would be given a piece of dried umbilical cord mixed with some banana to swallow. … My brother's wife didn't conceive for five years after their marriage and, somebody told to my brother's wife to swallow umbilical cord with the banana.

Although I never met anyone who claimed to have done this herself, second-hand accounts (‘my brother's wife’; ‘my neighbour's sister in law’) made appearances in most conversations I had across a range of mothers, doctors and bankers in Chennai. This belief and practice were reported as ‘common knowledge’ time and again by women I interviewed across Chennai's social and economic spectrum. Some women connected this practice explicitly to the more recent phenomenon of cord blood banking. In the words of one woman, ‘Women who are childless for many years, they are fed this umbilical cord along with banana. Now doctors have also started to preserve this’. Two obstetrician-gynaecologists narrated a case within the more coercive context of the pressures childless women face:

Doctor 1: Once a patient came to the clinic. She had taken [eaten] a bit of cord.

Doctor 2: They made her swallow it.

Doctor 1: To consume.

SH: And then?

Doctor 1: She had vomiting –

Doctor 2: But not due to pregnancy! No, no … somebody *forced* her to take it. She was upset. She came to us for infertility.

The spectre of infertility places women in a very vulnerable situation. Widespread across India are the linked practices of dowry and exogamous marriage. Brides' families must generate sizeable dowries in order to secure a suitable groom. Upon marriage, wives must produce a child in order to consolidate the security of their marriage. Women who fail to conceive within a year of marriage are at risk of violence and/or the humiliation of being returned to their natal family as ‘defective goods’ (often *sans* dowry). As a result, individuals' fertility in particular and fertility treatments in general are huge subjects of conversation, and generally begin at home.

Additionally, many I spoke with in Chennai reported a second ‘health insurance’ use for cord. Many women offered accounts of how amulets (*taayattu*), filled with one's own dried cord stump, are given to children to wear in the belief that the amulet wards off ill effects that little demons and devils (*karru*, *karuppu*) might have on the child. In the words of another woman, ‘I was told by my mother-in-law that they put it on the baby so nothing happens to the child. … It wards off evil spirits and the child will be fine, but it has to be [worn] on the child's body’. Similarly, another woman reported: ‘I have heard about keeping umbilical cord inside a small *dollar* [*taayattu*, amulet] made in gold and worn on a chain around the neck … so that the child will be safe’.

Cord blood marketers reported that prospective clients easily grasp the basics of the potential value of stem cell science and speculative finance, and that they do not regard these scientific and supernatural registers of value as mutually exclusive. Marketers report that, in their conversations with families, they often encounter these additional beliefs about the usefulness of umbilical cord and cord blood. One mother (also a private banking client) explained:

I think cord blood has cells or tissue that can be cultured and used for various organs – that much I know. … I think that is the most reproductive part of the cells. And it can be used some kind of re-generating on your organ, the blood can be used for regeneration. … I think my mother still preserves [my cord], but I do not know how usable it would be if it is just kept anywhere and everywhere. As far as I know it should be stored under certain conditions. I don't think it's reused; it's just a belief. But yes, still she keeps it.^15^

This tallies with how obstetricians characterized their patients: ‘Chennai patients are an incredible combination. On the one hand, they may have the most socially conservative practices that are deep-rooted in local traditions. On the other hand, they are totally scientifically savvy, they can tell you the very latest about organ transplants'. A cord blood stem cell bank employee explained, in similar terms, the relationship between cultural beliefs about the latent power of the placenta and umbilical cord and the ‘scientific miracle’ of stem cells as a coalescence of value:

People often tell us how the older generations used to have that piece kept for some time, that they would not throw it out – there must have been some powers that it was actually giving the baby or the mother – but you know, we really don't know what it was. And today it's really enlightening to know: yes, there *is* something there and we are getting it in research.

In her view, scientific value annexes and ‘repurposes’ stem cells' other value. Like this, many marketers report that prospective clients suspect that the supernatural properties of umbilical cords are likely to amplify stem cells' scientific and financial value.

## Interruptions

Nevertheless, this additional understanding of placental and cord power complicates bankers' narrative regarding umbilical cord blood as purely discarded disembodied biological matter; as ‘just waste’. Whereas potential clients' beliefs about umbilical cord's supernatural properties can underscore marketers' narratives about cells' scientific value, these beliefs can also undermine these narratives' value, to the extent that prospective clients decide against banking cord blood. One marketer explained: ‘Some of the people … in Tamil they'll say that they need to give [the cord] to the temple, to the god. They'll tell like that’. When I asked him what he would say in response, he replied: ‘In those situations, there is nothing that one *can* say. If the science does not speak to them, what could I possibly say [to persuade them to bank]?’ Another marketer described how the ‘old people’ in a family often raised concerns during her home visits about the advisability of allowing the cord to pass to someone outside the household: ‘Old people … the in-laws, the father-in-law, mother-in-law – they will ask and all and they will say, “We used to do that, we used to keep the cord in a *dollar* [amulet] and now you are asking me to keep it somewhere *else*?!” and they may not like it’. Bankers recognize that they must market not only to prospective parents, but also to prospective grandparents. As part of their marketing pitch they claim that cord blood not only has the potential to treat childhood conditions, but one day may be used to treat acquired conditions like diabetes and heart disease (now reaching epidemic proportions among India's elderly). Nevertheless, marketers and parents explain how older generations in a family regularly insist that umbilical cord's multiple qualities– scientific *and* supernatural – be considered before deciding to bank.

One cord blood stem cell banker narrated an instance of a failed collection:

The husband and wife had actually agreed to the whole process of cord blood banking and we had sent the nurse to do the sample collection. She was outside [the delivery suite], waiting to enter to collect the cord blood, and at that stage, the husband's father or mother in the *uur* [home town], rang up and said: ‘No. You cannot give your cord blood’. When [the nurse] asked the reason why, the parents explained that the grandparents insisted: ‘We don't know what they're gonna do with it! They should not do *anything* with it!’ *Athu yellam yenna panna porunga athu blood-odu. Uur-le yellam inda maadiri wothakku martunga* [No one will accept this sort of thing in our place]. The grandparents put their foot down so the nurse had to return without collecting.

This cord blood stem cell banker went on to narrate another case of non-collection in order to make the reasons behind this non-collection more explicit: ‘There were some other people who actually said of [cord blood] bankers: “*Avunga yetho oru manthiram yevenna panithiruppanga* – *Vendam*!” [They will use the cord in some or other sort of spell. No way! We do not want this]’. Potential clients' scepticism and distrust of cord blood stem cell banking was echoed by another doctor who explained that many of her patients hesitated before making a decision about banking their cord blood:

When I mention cord blood banking in the consultation, immediately they will say: ‘We need to consult the parents or in-laws’. Then I tell them to see the internet and absolutely all your doubts will be cleared there. This all patients do. Sometimes even after seeing the internet, the parents or in-laws say ‘No’. Because the idea is that people can do black magic with it, that the placenta should not fall into the wrong hands.^16^

Doctors and bankers report that prospective clients' families forgo the interlinked bio-medical and financial promise of stem cells once they begin to doubt the ability of biomedical expertise to cope with these cells' power. Here, families understand the coalescence of different registers, but consider that this coalescence creates potential value too potent to hand over. For these families, this very potency also calls into question the motives of bankers. With echoes of what one doctor narrated above regarding worries over possible black magic, one cord blood stem cell banking marketer reported that sometimes families ask him: ‘If it is so very valuable, then why should we give it to *you*?’ Constituted within this rejection of banking is the simultaneous rejection of cord blood stem cell bankers' narrative that cord blood is a ‘waste’ product.

## Accumulation after interruption

India has emerged in the scholarship on globalization, and health more generally, as a bit of a trickster figure (Cohen, 2011; Glasner, 2006; Sleeboom-Faulkner, 2011). At once shiny but also sinister, India appears in one guise as the health-care destination of choice for the budget-conscious patient (Woodman, 2007). A few mouse clicks away, India is an ‘organs bazaar’, a home to rent-a-womb surrogacy agencies and site for cost-effective, ethically suspect clinical trials. Nevertheless, in India the capital accumulation of health care surges ahead (Baru, 1998; Bharadwaj & Glasner, 2009; Patra & Sleeboom-Faulkner, 2011; Rao, 1999, 2009; Scheper-Hughes, 2000). My cord blood material shows how narratives of interlinked familial and financial ‘accumulation’ function to underwrite the relatively recent uptake of umbilical cord blood banking in Chennai. Although the corollary series of interruptions points to how these narratives can be rendered precarious, interruptions do not effect an overturning of this push towards accumulation. Instead, these interruptions form a key part of the constant re-constitution of narratives of accumulation (Laclau, 1990, pp. 89–92). While some prospective clients raise concerns on the way to deciding against banking, according to marketers, among those who raise doubts most do eventually bank. This is particularly the case, bankers explain, once ‘the science speaks to them’. The interruptions to cord blood banking in Chennai point out the tensions within, but ultimately serve as prompts for, the constant reiteration and thereby consolidation of ways in which the body has become enmeshed in multiple practices for the extraction and accumulation of wealth. In the final assessment, accumulation is as central to our understanding of the body as it is for our understanding of globalization.

## Notes

1 Nevertheless, both the Royal College of Obstetricians and Gynaecologists and the American College of Obstetricians and Gynecologists have raised questions about the utility of private cord blood banking on the ground that it is impossible to estimate any likelihood of an individual using her own stored cells (American College of Obstetricians and Gynecologists, 2008; Royal College of Obstetricians and Gynaecologists, 2006).

2 Unlike embryonic stem cells that have the ability to become many different kinds of cells in the body, stem cells found in umbilical cord blood are *blood* stem cells and can only replace other blood cells.

3 Cord blood is harvested immediately after birth by a nurse or other technician and then taken to a high-tech facility where the stem cells are removed and then stored cryogenically (frozen).

4 To date, all privately banked cells that have been used for treating blood disorders are those that have been harvested from a ‘saviour’ sibling's cord blood (American College of Obstetricians and Gynecologists, 2008; Royal College of Obstetricians and Gynaecologists, 2006). Generally, cord blood cells have been used in lieu of bone marrow transplants. Compared to bone marrow, cord blood harvesting is less invasive, easier to ‘match’ and more likely to ‘take’. A ‘saviour’ sibling is a child who is conceived (using assistive technologies to make sure the sibling is free of the disease) and born to provide an organ or cell transplant to a sibling who is affected with a fatal disease. Banked stem cells currently have a ‘shelf life’ of around 25 years.

5 Cord blood transplants do not have to be as close a genetic match to work as bone marrow transplants. Nevertheless, they still need to be quite close. The language used to explain the genetic ‘matching’ is that of HLA (human leukocyte antigen) markers. In bone marrow transplants, six of eight HLA markers must match. In cord blood transplants, four of six markers must match. See http://marrow.org/Patient/Transplant_Process/Search_Process/HLA_Matching_Finding_the_Best_Donor_or_Cord_Blood_Unit.aspx (accessed 20 December 2012).

6 One of the city's most prominent stem cell transplant haematologists estimates that she performs 30 to 40 non-experimental transplants annually in Chennai (other transplant haematologists perform experimental treatments).

7 Wider comparative national banking data are hard to come by, particularly for privately banked cells. As of August 2012, one of Chennai's largest cord blood banks' website claimed 46,000 samples banked. A cord blood banking executive director claimed that ‘[t]he stem cell story in India significantly lags [behind] other Asian and Western countries … only 15,000 clients have their children's stem cells preserved every year in India. This pales in comparison to China, Europe and the US which each report 100,000 clients on average’ (Knowledge@Wharton, 2010). One report estimated that, in 2008, China had banked between 70,000 and 250,000 samples (Technopolis, 2009, p. 12).

8 The term ‘public’ in public cord blood banks does not refer to state ownership; like most tissue or blood banks or organ registers, public cord blood banks maintain a register of searchable samples, open to the medical community. As with other kinds of public banks listed above, samples are bought and sold between banks, researchers and physicians, and banks charge processing and administrative fees.

9 This firm is trying to develop techniques for expansion of a blood sample (and with it a corollary increase in number of stem cells).

10 Here ‘mouse’ refers to an experimental animal, in contrast to the experimental treatments (for human patients) available at some of Chennai's corporate hospitals.

11 Skloot writes: ‘Henrietta [Lacks] died in 1951 from a vicious case of cervical cancer. … But before she died, a surgeon took samples of her tumor and put them in a Petri dish. Scientists had been trying to keep human cells alive in culture for decades, but they all eventually died. Henrietta's were different: they reproduced an entire generation every 24 hours, and they never stopped. … Her cells were part of research into the genes that cause cancer and those that suppress it; they helped develop drugs for treating [a vast range of diseases]. … Like guinea pigs and mice, Henrietta's cells have become the standard laboratory workhorse’ (2010, pp. 3–4). According to Skloot, at Johns Hopkins Hospital, where Lacks was treated in a segregated ward, she was never asked by the doctors for consent to donate her cells, nor was her family subsequently ever given a part of the proceeds that the sizeable trade in HeLa cells has generated over the decades since her death.

12 Retrieval incurs an additional cost.

13 I do not have information regarding the reason behind this significant drop in cost.

14 An extraordinarily detailed documentation of Indian beliefs and practices surrounding the placenta and umbilical cord (including those I list) is to be found in Ghurye (1963, pp. 110–14) in which he documents beliefs and practices found in Tamil- and Telugu-speaking south India. Although Ghurye carried out his research over a half-century before I carried out mine, many women I interviewed referred to their parents' and grandparents' beliefs (contemporary with Ghurye's research). Thanks to Mary Rader for help with sourcing this reference.

15 This interview was conducted by a research assistant, V. Padmavathy.

16 Here, ‘black magic’ is used as the standard English translation for *sooniyam*, a relatively commonly used term for ‘activities causing harm through evil spirits [*pey*, *pisasu*] by using a victim's hair, nail, foot, dust, etc’ (Cre-A, 1992, p. 467). The term ‘black magic’ was used regularly in my English-language conversations with Tamil speakers who were explaining others' anxieties about cord banking. In particular, as one obstetrician put it, the placenta (and cord) is an ‘organ of importance’, and, by the same token, banking raised concerns about the prospect of cord ‘falling into the wrong hands’. See also Ghurye (1963, p. 114). Although, compared to ‘science’, ‘black magic’ is generally regarded as a ‘lower’ form of accounting for and manipulating the workings of the natural world, it is also regularly reported on in India's mainstream media (e.g. ‘Spread awareness against black magic: The High Court directs the state government to spread awareness to curb the violence arising out of witchcraft and black magic’, *The Hindu*, 18 May 2012, p. 3; also ‘The former Chief Minister B. S. Yeddyurappa on Monday charged the former Prime Minister H. D. Deve Gowda and his sons with resorting to “black magic” to finish him off. “I am facing a threat to my life. I know the places where they did pujas under black magic. I will write to the Home Department on Tuesday complaining against the black magic of Mr. Gowda and his sons. They will be responsible if anything happens to my life,” he said. He said he would also write a public document in this regard’, *The Hindu*, 20 November 2007, http://www.thehindu.com/todays-paper/article1951565.ece.
